# Feasibility study applying a parametric model as the design generator for 3D–printed orthosis for fracture immobilization

**DOI:** 10.1186/s41205-017-0024-1

**Published:** 2018-01-11

**Authors:** Jianyou Li, Hiroya Tanaka

**Affiliations:** GGraduate School of Media and Governance, Keio University, 5322 Endo, Fujisawa-shi, Kanagawa 252-0882 Japan

**Keywords:** 3D–printing, Orthosis, Parametric modeling, Fracture immobilization, Customization

## Abstract

**Background:**

Applying 3D printing technology for the fabrication of custom-made orthoses provides significant advantages, including increased ventilation and lighter weights. Currently, the design of such orthoses is most often performed in the CAD environment, but creating the orthosis model is a time-consuming process that requires significant CAD experience. This skill gap limits clinicians from applying this technology in fracture treatment. The purpose of this study is to develop a parametric model as the design generator for 3D–printed orthoses for an inexperienced CAD user and to evaluate its feasibility and ease of use via a training and design exercise.

**Results:**

A set of automatic steps for orthosis modeling was developed as a parametric model using the Visual Programming Language in the CAD environment, and its interface and workflow were simplified to reduce the training period. A quick training program was formulated, and 5 participants from a nursing school completed the training within 15 mins. They verified its feasibility in an orthosis design exercise and designed 5 orthoses without assistance within 8 to 20 mins. The few faults and program errors that were observed in video analysis of the exercise showed improvable weaknesses caused by the scanning quality and modeling process.

**Conclusions:**

Compared to manual modeling instruction, this study highlighted the feasibility of using a parametric model for the design of 3D–printed orthoses and its greater ease of use for medical personnel compared to the CAD technique. The parametric model reduced the complex process of orthosis design to a few minutes, and a customized interface and training program accelerated the learning period. The results from the design exercise accurately reflect real-world situations in which an inexperienced user utilizes a generator as well as demonstrate the utility of the parametric model approach and strategy for training and interfacing.

## Background

Integration of 3D printing and medical image capturing technologies has been widely applied in medicine. CT or MRI anatomic imaging techniques can capture volumetric images of bones and soft tissues, and these images can be materialized by 3D printing devices as physical models to aid surgical planning or training [1–3]. Moreover, non-contact scanners based on a laser source and depth camera have become an option to replace the use of a conventional physical casting to acquire the anatomic surfaces necessary for the fabrication of orthoses or prosthetics. The 3D scanning technique prevents patient discomfort and induces less distortion of the target region [4, 5]. In addition to representing the anatomic form, the 3D printing technology also provides various physical properties to support the specific requirements of implants, orthotics, braces and prostheses via materials or structures that are built by manual or computational design [6, 7].

The same technology has been applied to fracture immobilization of the upper limb, and related studies have rapidly increased in recent years [8–14]. The 3D–printed orthosis minimizes distortion during the healing process because of its best fit geometry. The highly ventilated structure provides hygienic benefits and is light weight, reducing the risk of cutaneous complications and potentially improving treatment efficacy and increasing patient satisfaction [8]. The process of making a 3D–printed orthosis primarily consists of 3 digitalized phases [4], and studies have mainly focused on the 3D scanning stage to increase the precision and completeness of anatomic image acquisition of the affected limb [15–19]. Reviews related to the 3D printing stage are mainly devoted to the comparison of material appropriateness, fabrication technology and manufacturing efficiency [14, 19], and several groups have reported a 3D modeling process of wearable, ventilated and lightweight orthoses [8, 9, 12, 13]. In the 3D modeling stage, the design task is not only to generate a patient-specific shell according to the surface of the affected limb but also to control the density and thickness of the ventilated structure based on the surface. The structure and its volume impact the orthosis strength and printing time. Additionally, the necessary wearable designs, such as flexible gaps, hinges or interlocking components, are generated at this stage as well. It is often a challenge for the clinician to achieve initial treatment, design, and modeling steps in a 3D virtual environment, and this challenge includes the required time for orthosis modeling and the significant learning period necessary to utilize the specific CAD tool.

Currently, commercial CAD software is the main tool for researchers and designers to interface with the scanned anatomic mesh, build the orthosis model and export fabrication data [8]. The CAD tool provides complete commands and a 3D environment for researchers to explore the process of orthosis design; thus, they can develop stable command sequences as operable instructions for clinicians to reference. Such exploiting processes can be classified as Direct Modeling, an emerging technical term in the CAD industry [20–24] that has appeared frequently, in contrast to Parametric Modeling, for almost a decade. This flexible modeling process means that the user has significant freedom to compose and modify the geometric model directly without considering build history and parent-child relationships between features [21, 23].

**Fig. 1:**
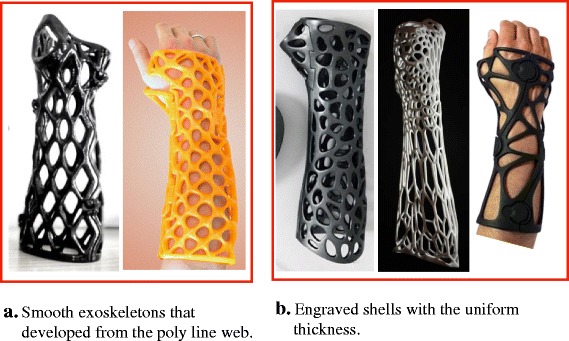
Different modeling approaches for orthosis prototypes. **a** Smooth exoskeletons. **b** Engraved shells

Based on increasing numbers of approaches and prototypes, Direct Modeling approaches have provided many successful results. The modeling procedure for 3D–printed orthoses has become an execution of steady and continuous tasks, and several common steps have emerged frequently from various approaches, such as cutting, thickening surface, and engraving shell [9]. The possible modeling process of an orthosis can be predicted from orthosis features (Fig. 1). For example, for Activarmor and Zdravprint splints (Fig. 1a) [25, 26], their spongy bone-like structures can be developed from the poly-line network along the limb surface, and the mesh appearances can be smoothed by the Catmull–Clark algorithm or T-spline surface technique [27]. Other prototypes have uniform thicknesses and obvious edges around their holes, such as the XKELET (Xkelet) or Cortex, designed by Jake Evill and Denis Karasahin’s Osteoid, (Fig. 1b) [28–30]; their common processes may include partial surface thickening as a solid shell and engraving the lattice by cutting or by Boolean operation. Although the orthosis model generation procedure can be archived from the above sequence, there is still concern regarding whether the clinician can learn and apply these modeling skills and perform them in clinical fracture treatment.

In many studies, the CAD software has generally received a negative evaluation based on its cumbersome interface. Because the software is designed for constructing multifaceted geometric forms for manufacturing or architecture purposes, the interface displays all icons, panels and information for constructing different embryos in the initial stage. Additionally, in the modeling process, each software has its own culture for providing requests and feedback in the interactions with the user. The user should be familiar with the system’s communication scheme and understand the related geometric principles and meaning of errors. Even for beginners in design or engineering schools, the learning period for the CAD tool usually involves weeks to months. In addition, although orthosis modeling is a fixed procedure, many variables change and impact the orthosis design in each individual design execution, such as the scanning quality, physiologic differences of anatomic limbs and fracture conditions. The clinician needs to react to these changes during modeling by modifying the immobilization region or lattice density, and these necessary reactions challenge the stability of fixed procedures. If the orthosis model is not printable or fails based on a geometric error, the clinician must have enough geometric knowledge and skill to solve the situation. The revised solution will probably require more time and be more complex than the modeling procedure itself.

Therefore, the developed modeling procedure is not currently suitable for provision as operable instructions for clinicians. The required time for these procedures usually ranges from tens of minutes to 3 h, depending on the operator’s skill [8, 9]. In such long operations, the complex interface and system interactions may cause clinician to fail to obtain a printable design. These attempts at orthosis modeling belong to an exploratory process that should only performed by a CAD expert for study purposes; for clinical treatment, this should be shifted to a teachable skill and an efficient tool for medical personnel in a medical context.

Relative to the Direct Modeling’s advantages for exploration, Parametric Modeling is suitable for fixed tasks to generate orthosis designs. In addition to managing dimensions, many modeling software programs can now edit complex parametric models via applications of text or visual programing languages to organize modeling steps, constraints and parametric relationships [31]. For example, Rhino 3D (Robert McNeel & Associates) works with Python or Grasshopper 3D, and Fusion 360 (Autodesk) works with a Dynamo plug-in. For reacting to variables in the orthosis design and generating stable results automatically in real-time, the orthosis modeling steps should be reconstructed by parametric modeling technology and become a history-based model. Orthosis features are well-generated by parameter-driven input, pre-defined algorithms and parent-child relationships of geometry.

In this manuscript, a parametric model and its customized interface were developed for clinicians to create printable orthosis casts with minimal CAD skill required. A training tutorial was formulated and evaluated by inexperienced users in an orthosis design exercise to determine its feasibility and ease of use.

## Method

**Fig. 2:**
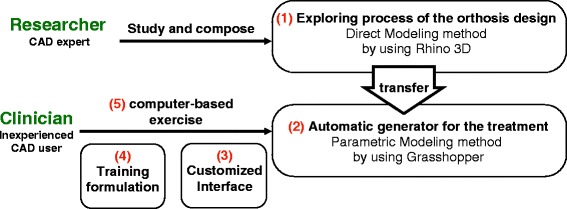
Development process for transferring the explored modeling process into the parametric model

In this section, we describe the 5 stages of transferring the results of a Direct Modeling approach to develop a practical parametric model (Fig. 2), as well as the volunteers who participated in the subsequent training and evaluation. The points of each stage are listed as follows:

- Direct Modeling process of orthosis design: A compact process developed based on the clinician, the inexperienced CAD user’s thinking and limited geometric knowledge.
- Parametric model: Reconstruction based on the results of previous stages; the main parameters in every step were collected and optimized in iterative tests.
- Interface customization: All unnecessary menu, toolbars and panels in the main CAD environment were removed, until only a basic interface remained.
- Quick training: Fundamental knowledge for utilizing the parametric model was provided in a one-on-one tutorial, including viewport navigation, poly-line drawing in Rhino and object setting in Grasshopper. Five nursing students who were familiar with manipulating fracture immobilizations were invited to participate in the training.
- Orthosis design exercise: After the training, the participants performed a computer-based exercise to design orthoses for 5 different arm models by themselves, and their design processes on the screens were recorded for further analyses.

### Scanning process

**Fig. 3:**
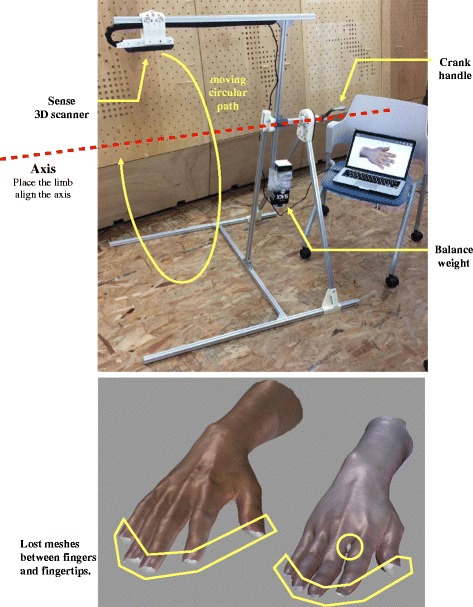
3D scanner mount and scanning limitations

Although the 3D scanning process was not the main point of this study, the scan quality and other limitations may impact the stability of the parametric model. Based on the literatures [9, 17], patients with acute fractures may have difficulty maintaining required poses, or the clinician may be unable to move the scanner to all the required positions around the limb stably. To develop a complete mesh model of an affected limb, we utilized the 3D scanner Sense (3DSystem) and a custom-made scanner mount (Fig. 3a) to collect limb samples. The scanner mount was made by aluminum rail sticks and the scanner was installed on its rotor arm. A support table was placed under the mount’s axis, and the patient’s upper arm for the affected limb was supported to align with the axis. The scanner on the rotor arm could be rotated and moved along a circular path around the limb smoothly by rotating the crank handle and was maintained at a stable distance from the limb. The entire scanning process took approximately 20 s. However, the scanner always faced the axis line in the process. A few detailed faces between the fingers and fingertips could not be reached (the yellow area in Fig. 3b), and in subsequent definitions of the immobilization area, the operator should avoid these areas because these lost regions may lead to subsequent failure of the modeling command.

### CAD software and parametric modeling tool

Considering the fitness and adjustability of the surface-based model, we utilized Rhino 3D Version 5 for the main modeling environment; this is recognized as a typical non-parametric software. Hence, it has the flexibility of Direct Modeling, and can address the simultaneous existence of mesh and free-surfaces. Additionally, it allows the user to customize the interface and remove all unnecessary panels and tool bars. Moreover, its algorithm plug-in, Grasshopper 3D, is a widespread Visual Programming Language among parametric modeling tools [31–34], and it is complementary to the flexible property of Rhino 3D. In the steps described below, we utilized Rhino 3D to simulate the modeling sequence directly and transferred it to an automatic parametric model via the corresponding components (graphic icon showing the program command) in Grasshopper 3D.

### Automatic arrangement

**Fig. 4:**
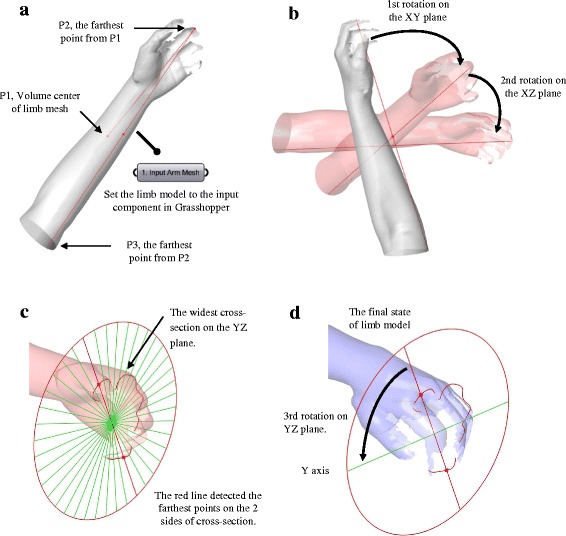
Working process of automatic arrangement in Grasshopper. **a** Import the limb model to Grasshopper. **b** Rotations on the XY and XZ planes. **c** Widest cross-section on the YZ plane. **d** Rotation on YZ plane

When importing the scanned limb, the mesh model may appear in the Rhino 3D space with random angles and positions; therefore the clinician may need to move it to the appropriate position and rotate it to the right angle before moving forward. To save the clinician from having to learn these commands, as well as reducing the operating time and the risk of the model becoming lost in CAD space, we developed an intelligent function for automatic arrangement in Grasshopper (Fig. 4). Once the limb model is set to the mesh component and the model data are imported to Grasshopper, the arrangement function can determine the mesh volume central point P1, and locate the 2 mesh central points, P2 and P3, on the two ends of limb to form the P23 axis (Fig. 4a). Then, the angles between the axis line and 2 planes, XY and XZ, are determined (Fig. 4b), which provide automatic rotations in each plane to allow the limb to align with the X-axis. A set of cross-sections on this arm will be determine by circles on 30 planes along the P23 axis, and the widest section curve is usually located on the palm (Fig. 4c). The two farthest points on this section can be found using the cross-points from an array of 40 lines. The angle between this line and the Y-axis could be applied to the final rotation on the YZ plane, with palm facing up or down. Then, the arm model is moved from P1 to a default position on the coordinates (300, 100, −50). The clinician can then define the immobilization area on this position using the top view clearly.

### Parametric modeling process

In the explorative process of Direct Modeling, all adopted steps should have their corresponding commands in Grasshopper and be transferable to the parametric model. However, a few steps are only operable in the programming language, e.g., the Voronoi pattern for the engraving operation is difficult to generate manually in Rhino. Therefore, the process of parametric modeling is more complete; we have described its detailed steps directly and explained the related calculations in the program.

**Fig. 5:**
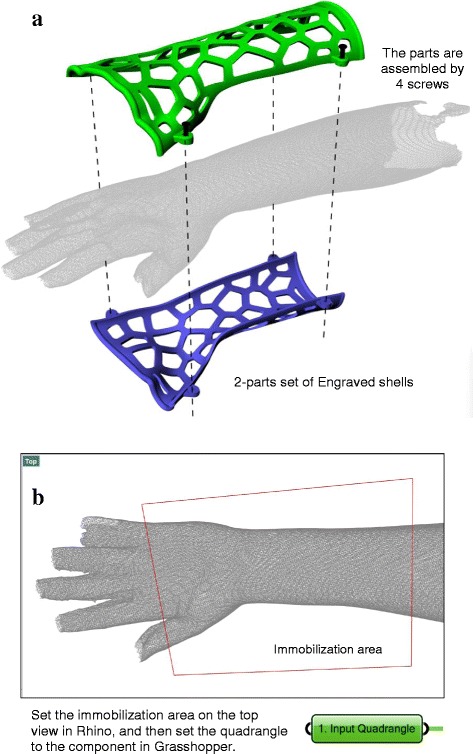
Features of orthosis in this manuscript and its defining method for immobilization area. **a** Assembly method. **b** The defining method for immobilization area

**Fig. 6:**
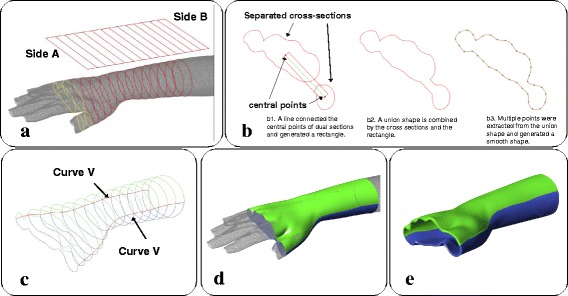
The process of merging multiple cross-sections, generating covering surface and thickening. **a** Multiple cross-sections appear between the palm and thumb. **b** Merging procedure of the cross-sections. **c** Extreme points on all cross-sections and V curves. **d** 2 separate surfaces are generated by the V curves and cross-sections. **e** Thickening operation

First, the 3D–printed orthosis described in this manuscript consisted of 2 pieces of engraved shells fastened by 4 screws (Fig. 5a). The method to define the immobilization area involved drawing a quadrangle (Fig. 5b). According to the patient’s condition, the clinician can use the poly-line tool to draw the quadrangle and overlay it on the affected limb in the top view in Rhino. Then, the quadrangle could be set with the Curve Component in Grasshopper 3D and input into the parametric model. When receiving input data, the program can recognize Sides A and B of the quadrangle by sorting the coordinates of their midpoints on the X-axis, as well as generate a set of lines between the 2 sides with the Tween Curve command (Fig. 6a). The number of lines is decided by the distance between the midpoints of Sides A and B, and the insertion of one line every 15 mm over this distance is predicted. In this case, the distance was 216 mm, and 14 lines were inserted. These lines were projected onto the limb model to obtain cross-sections.

However, if the quadrangle includes the gap between the palm and thumb, multiple cross-sections will appear on these projections (yellow curves in Fig. 6a). The presence of multiple cross-sections will cause the next Network operation to fail because only a single cross-section is allowed in each projection to generate the surface. Therefore, a procedure was designed to merge these cross-sections (Fig. 6b). When dual cross-sections were detected, a line will pass through the central points of the separate cross-sections. This will be offset on both sides as a rectangle, and a new union shape will be formed by the combination of the rectangle and the connected cross-sections. The union shape is then smoothed by extracting points from itself and regenerated a similar shape by the Interpolate Curve command. These union shapes will replace the multiple cross-sections, maintaining a single shape in each projection. Additionally, the design of this slim gap between the cross-sections can fix the location of the thumb for treatment demands.

Then, the extreme points on the Y-axis of all cross-sections were located and connected as two red curves (V Curves in Fig. 6c), and cross-sections were divided by the 2 curves into a green set and a blue set. With the V curves, these can form 2 separate surfaces (green and blue surfaces in Fig. 6d) via the Network command. The above sequence only took a few seconds to generate the surfaces as an initial result, and we subsequently visualized the immobilization area as a 3D surface. After the covering surface was generated, the limb display could be turned off to allow the clinician to evaluate the inside of the surface. If the covering surface fit the limb well, the clinician can then trigger the program to continue the thickening operation by offsetting the surfaces with a thickness between 3 and 5 mm (Fig. 6e), depending on the whole orthosis area.

**Fig. 7:**
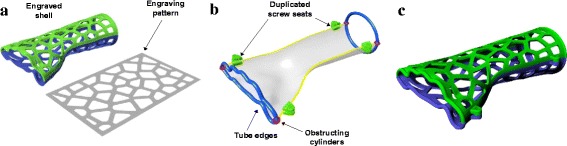
The process of generating engraved shells, screw seats and tube edges. **a** The engraving pattern and the engraved shells. **b** Positions of the screw seats and tube edges. **c** Combined result

For calculating the engraving pattern (Fig. 7a), a rectangle was generated with the pattern’s area - the width and length of the rectangle were determined based on the averages of the covering surface edge lengths. We applied the common Voronoi diagram for the engraving pattern. The amount of seed points of the Voronoi diagram is defined as between 40 and 80 and is directly proportional to the rectangle area. The pattern was mapped onto the inside and outside surfaces of the shell, and hollowed out for the holes. The last step was to develop screw seats and tube edges to increase the orthosis’ ability to be worn (Fig. 7b); these were subsequently combined with the engraved shells (Fig. 7c). A screw seat was embedded in the parametric model’s internal source and copied to the 4 positions on the V curves. The tubes were generated along the edges of the covering surface to provide a comfortable interface between the skin and the rigid shell, and 4 obstructing cylinders were used to separate the tubes and ensure that each tube only interlocked with one of the shells. All the tubes and screw seats were distributed and combined to their related shell by the Solid Union command, and the shells were then ready to be exported as a STL file.

### Workflow and customized interface

**Fig. 8:**
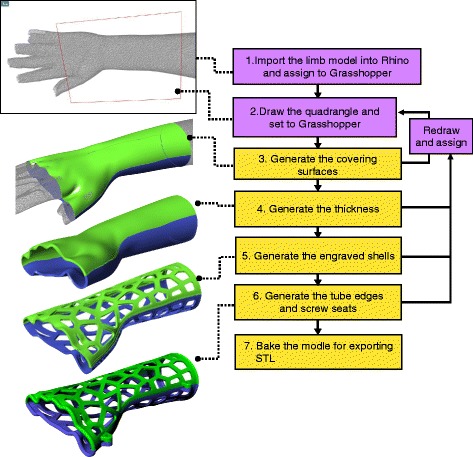
Operation workflow of orthosis design

From the above modeling procedure, the overall workflow of the orthosis design is shown as a flow chart (Fig. 8). This represents the exact process that the operator will face, and all detailed modeling calculations are working behind this interface. Steps 1 and 2 are in the input stage, and step 3 to 7 belong to the modeling calculation that is controlled by the operator. Considering that several clinicians may lack related fabrication experience with 3D printing, the main parameters in steps 3 to 6, such as the thickness and amount of Voronoi seed points, were optimized in iterative tests to archive a balance between basic strength and being lightweight.

**Fig. 9:**
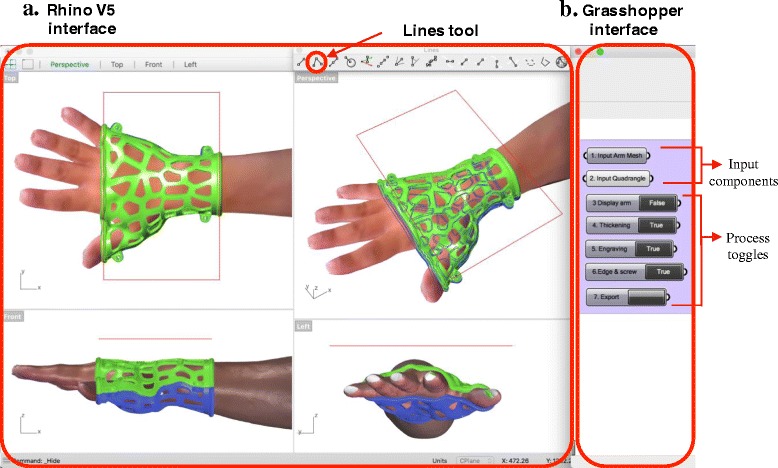
Customized interface of Rhino and Grasshopper. **a** Rhino V5 interface. **b** Grasshopper interface

Based on the workflow, the integrated interface of Rhino and Grasshopper was customized (Fig. 9), and it was simplified to reduce the learning period for the clinician. All menu, toolbars and panels in the Rhino interface were removed, and only 4 viewports and the Lines tool are required for clinician to draw a quadrangle and evaluate the model state visually (Fig. 9a). On the Grasshopper interface, the main node-based program and menu are hidden (Fig. 9b), and the clinician does not need to know how the program works. There are 7 necessary components, which are displayed and numbered according to the workflow. The first two components can receive the data from the limb model and the quadrangle by setting them in their component menus, and then generate the covering surfaces. The next 5 True/False toggles control the arm display, main data flow of the model generation and final model export, and Toggles 4 to 6 can output True values and an updated orthosis model in the order shown in Fig. 8, including thicken surfaces, engraved shells and final shells with screw seats and tube edges. Each step takes less than 10 s to update the model.

**Fig. 10:**
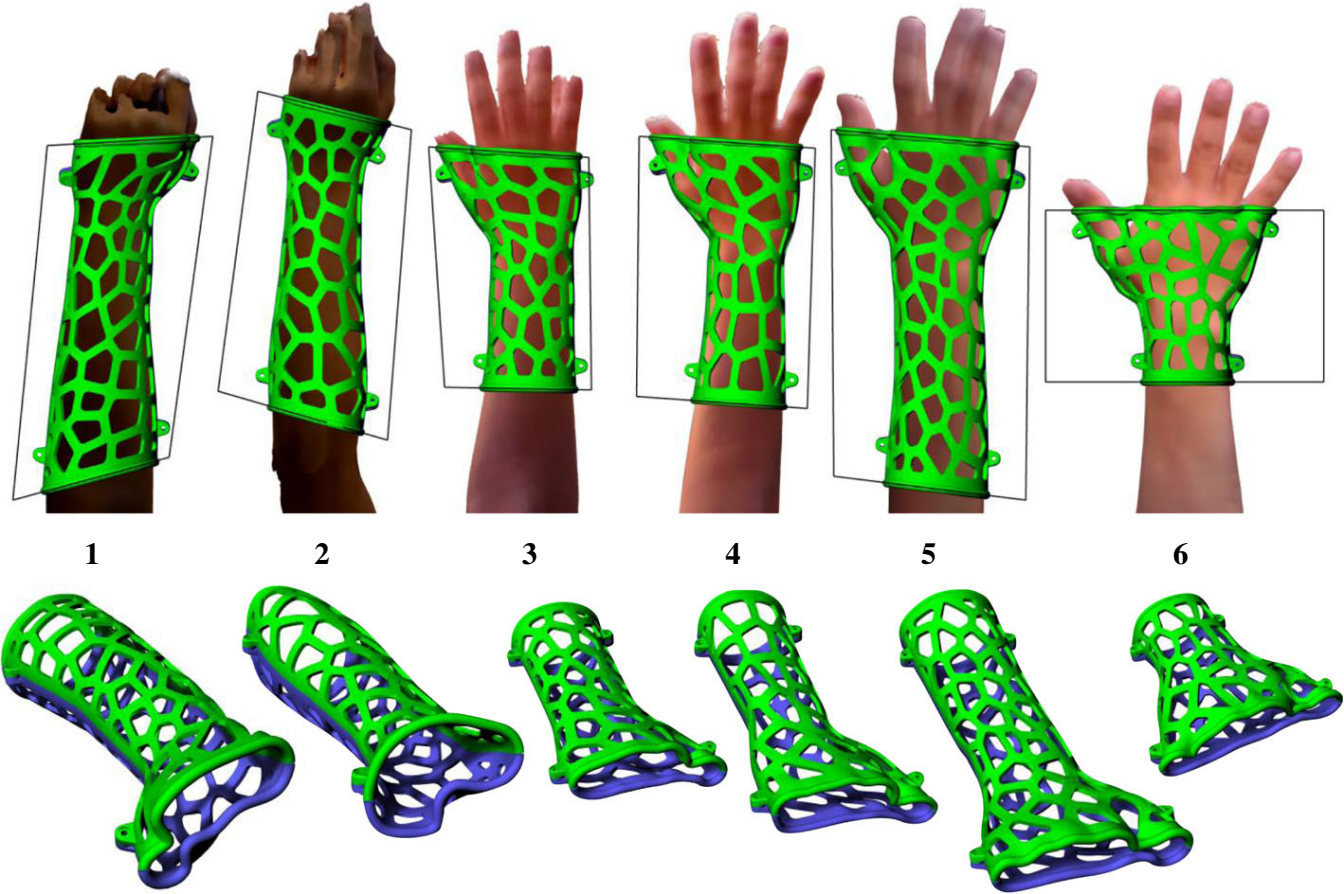
Stability test results for 6 different limbs

We collected 10 different anatomic models of the upper limb from adult volunteers, and then applied the parametric model to these arms to determine the program’s performance and stability with different mesh conditions. The optimization of the main parameters was also studied from these samples. 6 sets of limbs, orthoses and their immobilization settings are shown in Fig. 10. These limb models were also used in the following training and design exercise.

### Training

Based on the above modeling process, workflow and interface, a training program was formulated to teach clinicians to utilize this parametric model of orthosis design and export a printable model. The training content included an introduction to 3D–printed orthosis, an operating tutorial and computer-based practice. Five nursing students in their junior year were invited to undergo this training, and they then completed an orthosis design exercise to evaluate the function of the parametric model and training. The participants had internship experience in the orthopedic department in the hospital and were familiar with manipulating fracture immobilizations. They were capable of operating document software, internet browsers and apps on mobile device in daily life, but did not have any CAD background or 3D printing experience.

**Fig. 11:**
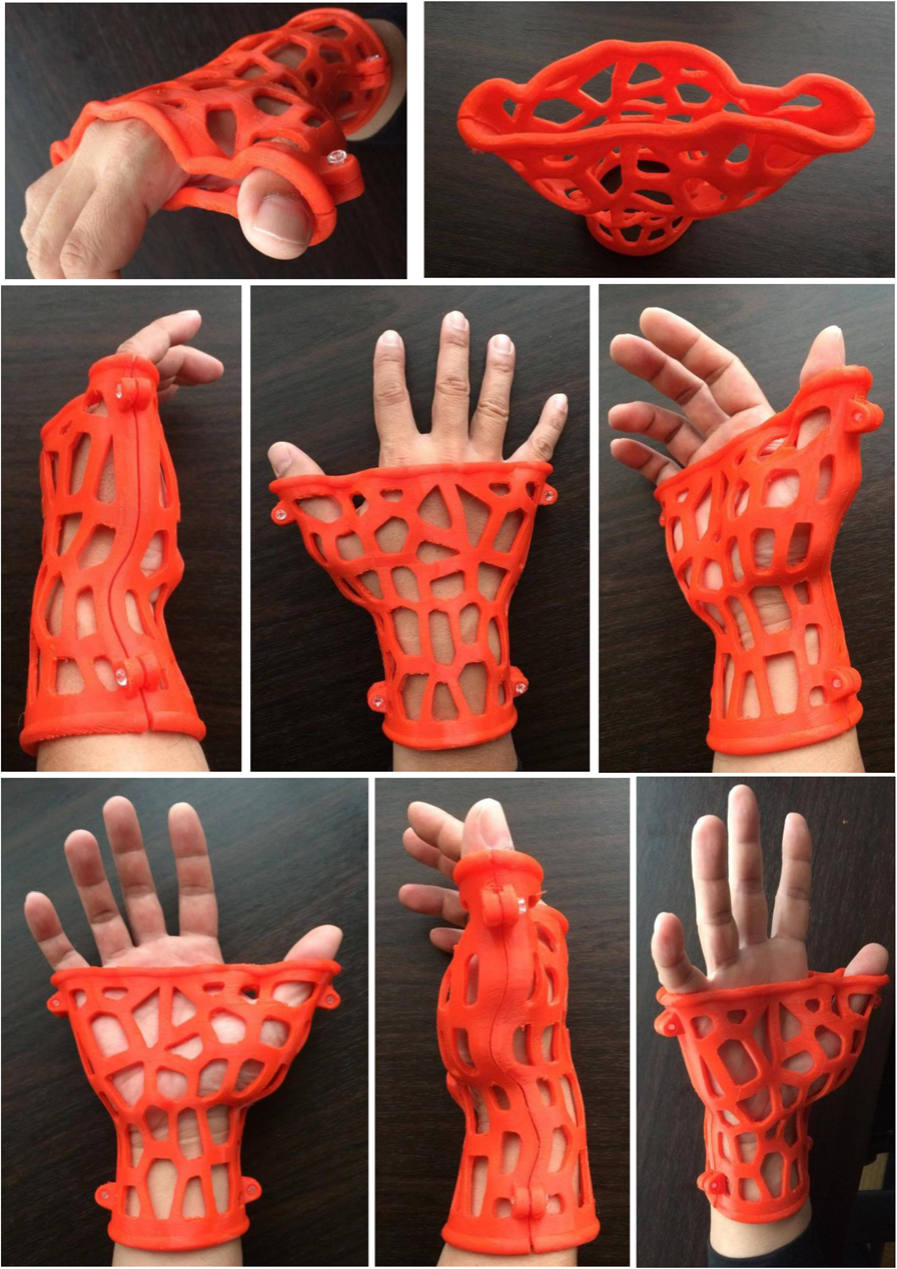
Physical orthosis prototype of limb sample 6

The introduction included demonstrations of the digital models and physical orthosis and explanations of the orthosis design and 3D printing process to the participants. One of the prototypes was prefabricated with a FDM printer Quditech1 (Qiditech) (Fig. 11) for demonstration, and this took approximately 3 h. The participant could learn how the 3D–printed orthosis was assembled, produced and functioned for patient rehabilitation. Then, the computer-based tutorial was provided one-on-one, and the necessary operational knowledge of the Rhino and Grasshopper programs was summarized as the following points:

- Basic viewport navigation in Rhino: The viewpoint operations include: zoom in/out, rotate view, pan move and switch viewport. These are basic skills necessary to identify the CAD space and evaluate the limb or orthosis models from any viewpoint freely.
- Draw and fix the quadrangle: The drawing is operated by setting 4 corner points of a quadrangle in the top view, and accomplished atomically when the shape is closed. If the quadrangle does not match the expected immobilization area, the operator can redraw it to replace a previous one.
- Select Rhino object and assign to Grasshopper: Selecting and setting objects are usually executed together in the input task. The operator needs to learn the select, cancel selection, and delete commands and the selected object state. After selecting the model or poly-line, the operator can input or clear setting contents in the input component menu in Grasshopper.
- Control data flow in Grasshopper: Clicking the toggles can change its output (True/False) and then send out the geometric data to next modeling process. The orthosis model will be updated by clicking toggles in order, and most toggles do not work if the previous toggle produced a false value.
- Solve program error or software crash: Sometimes, because the immobilization area overlapped on the limb model’s edge or a hole, the parametric model may generate a distorted surface, separate objects or have no response when attempting to update a model, even causing Rhino to crash. Correcting the immobilization area from the edge or hole can avoid these problems.

5 limb models were used during this tutorial. The tutor used 2 of these to demonstrate the process, and participants followed the same steps. The participants could ask the tutor to repeat the process until they had memorized the whole procedure and its underlying logic. Then, another 3 limb models were provided to participants for practice, and they were asked to design orthoses without the tutor’s help. The participants were encouraged to solve the problems that arose during the process by themselves as much as possible, but they could ask the tutor for hints as needed. The total time during the training was recorded after they accomplished the procedure.

### Orthosis design exercise

After the training, the participants completed a trial to design orthoses for another 5 limb models on their own. The limb models were saved in different layers of a file, and participants were asked to switch the layers and design the orthoses in order.

**Fig. 12:**
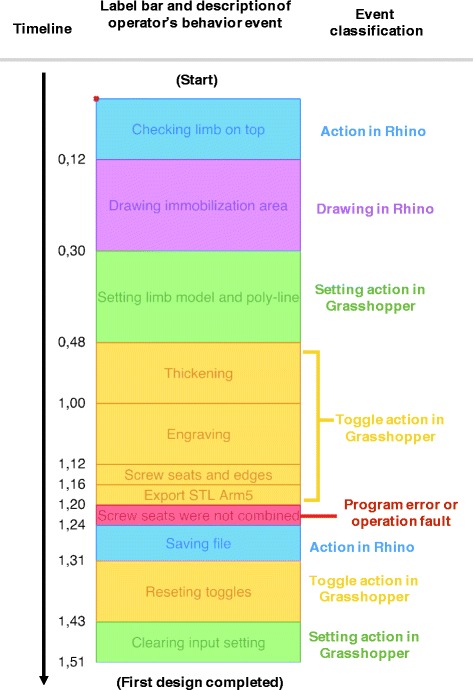
Visualized format of participant’s video record, labeled bar chart

The whole design process was recorded from the screen as video, and the video was monitored and marked with labels to identify every operator’s operational movement (event) on the timeline as a bar chart (Fig. 12). This sample chart shows the video record of an orthosis design process, a completed workflow that took 1 min and 51 s. We used 5 types of color labels to indicate the participant’s different purposes and working interfaces for each event on the timeline. The period of marking a label was initiated and completed according to the user’s mouse clicks and the working interface. The purple label represents the drawing immobilization line, and we determined how much time each participant spent on drawing to evaluate the difficulty of the task. The yellow label represents the waiting time after the participant clicked the toggles to obtain an updated model, and this was equivalent to the overall time of the modeling calculation. Program errors of the parametric model or operation faults are indicated by the red label, and the length of the red label represents the required time that participants needed to solve the situation and continue with the correct workflow. These labels helped us to visualize where and how the errors and mistakes occurred and the time expended on each event. Thus, we could improve the parametric model or training program.

After the participants completed the exercise, an interview was held. If the participant’s intention for any event was not obvious enough to determine a label, e.g., they were confused or forgot a step, the tutor should confirm what occurred with the participant in the interview. However, the main purpose of the interview was to collect the participants’ opinions regarding the parametric model and training program based on their experience.

## Results and discussion

A parametric CAD model for 3D–printed orthosis design was developed for clinicians who were inexperienced with CAD tools. The model utilized Rhino 3D V5, and its parametric program was constructed using Grasshopper 3D. We input 10 different limb models into this parametric model representing the patients’ affected limb and used different immobilization areas to generate a printable model. Overall, the parametric model was stable if no hole or edge was present in the immobilization area.

The parametric model’s interface and operation workflow have been extremely simplified to reduce the learning period required for the clinician. For evaluating the interface and parametric model performance, a training tutorial combining computer-based practice was created and held for 5 participants who were capable of executing conversional fracture immobilization. In the training, all participants followed the tutorial, realized the operation steps and accomplished practice within 15 mins.

**Fig. 13:**
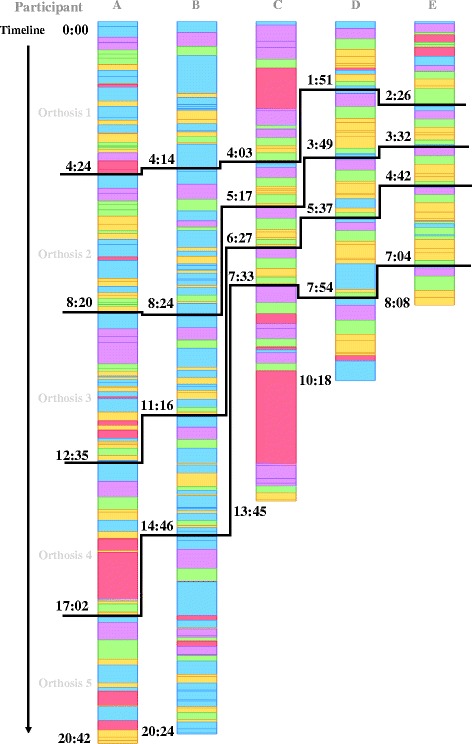
Labeled bar chart of 5 participants’ video records

Then, the participants completed an orthosis design exercise, and they were asked to complete 5 orthosis designs using parametric models on their own. Their recorded videos of the design process were labeled A to E and visualized using color labels as in the bar charts of Fig. 13. We differentiated each orthosis design period on the timeline from the other designs by black lines, and marked the precise time points when they finished each design, i.e., participant A finished the first orthosis at 4 mins and 24 s, whereas participant B spent 4 m 14 s. From the video visualization, the participants finished the 5 designs in a period ranging from 8 to 21 mins, and each design took between 1 and 7 mins. Compared to manual modeling, the time required for the parametric model has been reduced dramatically. From the interview, most participants gave positive evaluations of this digital tool and training, and more practice and verification with a printed model could help them avoid mistakes and improve their drawing skills to define the immobilization area.

In the video, we marked the following program error and participant mistakes with red labels.

- Wrong operation: The participant took unnecessary steps or missed an operation, such as moving the wrong objects or utilizing extra clicks, although these negative faults did not impact the process critically. We added these faults as frequently asked questions in the updated tutorial, which allowed other beginners to avoid repeating them.
- Input failure or invalid model generated: Usually these program errors occurred after the input setting or during the modeling calculation. The parametric model did not update the orthosis model after the toggles were activated, e.g., the thickening or engraving function failed, or sometimes it generated a valid model that had a distorted surface or separate objects on the shell. These errors indicated defects in the Grasshopper program or limb model.
- Software crash: If the participant wasn’t aware of the boundary or lost the mesh on the limb model, they may have set immobilization areas overlapping the model’s edge or a hole. The model process may generate incomplete cross-sections or geometries and cause Rhino to crash because they disturbed the data tree and initiated massive numbers of calculations instantly in Grasshopper. The participant needed to restart the software and modify the immobilization area again.

The frequency of red label marking was enumerated from 25 orthosis designs in Table 1. Only 2 crashes occurred, and the participants learned to modify the immobilization area to solved these issues by themselves. Very high fault times occurred in participant A’s record, although most faults were minimal, such as moving the wrong object or performing extra clicks absentmindedly. The faults did not obstruct the participant significantly, and the parametric model worked perfectly.

**Table 1:** Accumulation of red labels

	1. Operation fault	2. Input failure or invalid generation	3. Software crash
A	11	0	0
B	0	2	0
C	1	2	1
D	1	1	0
E	1	1	1

**Fig. 14:**
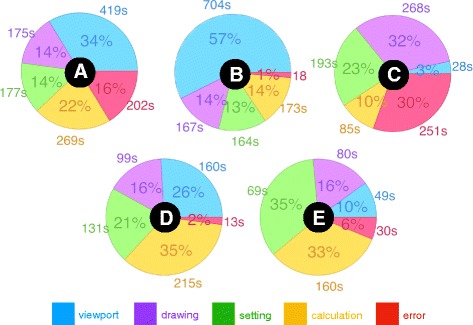
Pie chart of event percentages and seconds spent in the participants’ videos. The pie charts are marked A to E to present the participant labels

Additionally, several interesting discoveries from the video provided useful feedback regarding the participant’s behavior. We extracted the total time expended from the event labels as well as their percentages relative to the whole process for each participant, and presented them as a pie chart in Fig. 14. In the analyzed results of participant B, we found the participant made almost no mistakes in the operation during the exercise and no crash occurred. The blue labels represented 57% of her process; the observations were confirmed from the video, with a high blue percentage indicating she spent most of the time checking the limb model’s appearance and edges in the viewport and deciding how to draw the immobilization area. Her drawing avoided the edges and holes of the model skillfully, and this was the factor that allows the program to generate the orthosis model successfully. Long seconds of drawing were found in participant C’s process, and repeated drawing occurred in the video when the immobilization area did not cover the expected surface. Developing another input drawing method could solve this challenge, but this is usually limited by the available drawing command and necessary geometric logic to generate cross-sections. Additionally, long periods of waiting for software calculations also appeared in participant A and D’s videos, and long calculations usually occurred in the engraving pattern, especially when it worked on a deformed surface around the wrist. A hexagon or diamond array would be more stable than the Voronoi diagram for the engraving task. The random points of the Voronoi algorithm do marginally increase the risk of generating tiny holes and failing the projection on the surface.

## Conclusion

In this study, to address the relative advantages and disadvantages of Direct Modeling and Parametric Modeling, a design generator of 3D–printed orthosis and its detailed modeling process were created and developed by the Visual Programming Language in an engineering CAD environment. The interface and workflow of the parametric model were successful for use by clinicians who were inexperienced to CAD software.

In the evaluation of the feasibility of this digitalized tool, we received positive feedback from the training and design exercise in 3 target areas:

- Required period of beginner training: In the training, the simplified interface and workflow successfully reduced the required geometric knowledge for participants and the required training period to 15 mins.
- Operation efficiency for clinicians: In the orthosis design exercise, under the parametric model’s facilitation, the required time for executing an orthosis design was dramatically reduced to a few minutes.
- Performance stability of the parametric model: The frequency of software crashes and program errors was acceptable for 25 designs and can likely be improved with further study.

Finally, the process and results of the design exercise reflected the real-world situation of clinicians operating the parametric model for orthosis generation and also revealed unforeseen factors, such as participant operating difficulty, personal behavior and reasons for program errors or good performances. These discoveries provided a clear direction for improving the parametric model and training program and helped advance this tool towards a more practical level for medical applications.
